# Pickering Double Emulsions Stabilized with Chitin Nanocrystals and Myristic Acid-Functionalized Silica Nanoparticles for Curcumin and Chlorogenic Acid Co-Delivery

**DOI:** 10.3390/pharmaceutics17040521

**Published:** 2025-04-16

**Authors:** Javier Paredes-Toledo, Javier Herrera, Javier Morales, Paz Robert, Joaquín Gómez-Estaca, Begoña Giménez

**Affiliations:** 1 Department of Food Science and Technology, Faculty of Technology, University of Santiago of Chile, Av. Víctor Jara 3769, Estación Central, Santiago 9170124, Chile; javier.paredes@usach.cl (J.P.-T.); javier.herrera.c@usach.cl (J.H.); 2 Department of Pharmaceutic Science and Technology, Faculty of Chemical and Pharmaceutical Sciences, University of Chile, Santos Dumont 964, Independencia, Santiago 8380494, Chile; javiermv@ciq.uchile.cl; 3 Department of Food Science and Chemical Technology, Faculty of Chemical and Pharmaceutical Sciences, University of Chile, Santos Dumont 964, Independencia, Santiago 8380494, Chile; proberts@uchile.cl; 4 Institute of Food Science, Technology and Nutrition (ICTAN-CSIC), José Antonio Novais 6, 28040 Madrid, Spain; joaquin.gomez@csic.es

**Keywords:** Pickering double emulsion, in vitro digestion, co-delivery, silica nanoparticles, chitin nanocrystals

## Abstract

**Background/Objectives**: Double emulsions (DEs) enable the simultaneous encapsulation of water-soluble and oil-soluble bioactive compounds. Their stability can be enhanced through Pickering stabilization, where solid particles are irreversibly anchored at the interfaces, forming a steric barrier. This study aimed to evaluate the release behavior of curcumin and chlorogenic acid (CA) in Pickering DEs formulated with chitin nanocrystals (ChNCs) stabilizing the outer interface (DE-ChNC) and both ChNCs and myristic acid-functionalized silica nanoparticles (SNPs-C14) stabilizing the outer and inner interfaces (DE-ChNC-C14) under in vitro gastrointestinal digestion. **Methods**: The optimal homogenization parameters (time and speed) for stabilizing the external interface with ChNCs were determined using a statistical design. Pickering DEs were characterized (droplet size and size distribution, microstructure, creaming, encapsulation efficiency and stability, rheological behavior) and subjected to the INFOGEST digestion method. **Results**: ChNCs effectively maintained the droplet size, microstructure, and ζ-potential, preventing coalescence and creaming while enhancing viscosity and gel-like behavior, contributing to improved physical stability. The CA encapsulation efficiency was higher in DE-ChNC-C14 (91.4%) than in DE-ChNC (45.0%) due to the presence of SNPs-C14 at the inner interface, which improved CA retention during storage. CA was gradually released from DE-ChNC-C14 throughout digestion, with bioaccessibility similar to that of the control DE (stabilized with conventional emulsifiers; ~60%). Curcumin bioaccessibility in the Pickering DEs was relatively high (~40%) but lower than in the control DE, as the ChNCs reduced lipid digestion and curcumin bioaccessibility. **Conclusions**: ChNCs and SNPs-C14 effectively stabilized the outer and inner interfaces of DEs, enabling the simultaneous release of water-soluble and oil-soluble bioactives with health benefits.

## 1. Introduction

Double emulsions (DEs), consisting of a primary emulsion dispersed in a continuous phase [1], have attracted significant interest in several fields, including cosmetics, food, and pharmaceuticals. Water-in-oil-in-water emulsion (W/O/W) is the most studied type of DE in foods, as many foods consist of continuous aqueous phases [2]. A key application of DEs is the encapsulation of bioactive compounds, for example, antioxidant compounds, colorants, enzymes, peptides, minerals, probiotics, and vitamins [3]. Furthermore, thanks to the presence of two water phases and an oil phase, DEs allow the encapsulation of both oil-soluble and water-soluble bioactives in a single system (e.g., curcumin/catechin [4]; lycopene/epigallocatechin gallate [5]; xanthoxylin/vitamin C [6]; astaxanthin/phycocyanin [7]; β-carotene/riboflavin [8]). This unique structure also provides DEs with great potential as co-delivery matrices, as the water-soluble bioactives entrapped in the internal phase can be released in a controlled manner during digestion, while simultaneously enhancing the solubilization of lipophilic bioactives by forming mixed micelles during the hydrolysis of the oil phase of the emulsion [9]. However, their three-phase structure also renders conventional DEs stabilized with emulsifiers (surfactants and/or biopolymers) thermodynamically unstable and prone to numerous destabilization phenomena [3,10], which may limit their potential applications. These instability mechanisms can be further exacerbated under gastrointestinal conditions, where DEs are exposed to adverse factors such as mechanical forces, enzymatic action, surfactants, and variations in temperature, ionic strength, and pH [11]. In this context, Pickering DEs, in which solid particles stabilize one or both interfaces of the DE, have garnered growing attention in emulsion-based delivery systems over the past few years because of their higher thermodynamic stability and enhanced physical stability compared to conventional DEs [12,13,14]. In these DEs, stability arises from the non-reversible adsorption of solid particles at the interfaces, leading to the formation of a tight particle covering around droplets that avoids droplet coalescence and also prevents the loss of the inner phase [10,13]. Nonetheless, research on the structural integrity of Pickering DEs and the release of bioactives entrapped in their multi-compartmentalized structure under gastrointestinal conditions remains relatively scarce [10,13].

Most of the literature on Pickering DEs focuses on the study of stabilizing the outer interface (O:W_2_) with solid particles while using conventional surfactants (mainly polyglycerol polyricinoleate (PGPR) or Span) at the inner interface (W_1_:O) [13]. The current trend in Pickering stabilization of the outer interface of DEs is to use natural biopolymer-derived particles, which are preferably wetted by water. Polysaccharide particles (e.g., starch, cellulose), protein particles (e.g., zein, kafirin, gliadin), and protein–polysaccharide composite particles have been employed for this purpose [10,13,14,15,16]. In this context, it should be noted that, as far as we know, chitin-based particles (chitin nanocrystals and chitin nanofibers) have not yet been used for stabilizing DEs. However, several studies have reported the successful stabilization of simple O/W emulsions using chitin-based particles, providing them with high storage stability [14,17] and enabling the controlled release of hydrophobic bioactives during gastrointestinal digestion [18,19,20]. This suggests that they could also be suitable for stabilizing the outer interface of DEs and enhancing the performance of Pickering DEs under gastrointestinal conditions. Chitin nanocrystals (ChNCs) can be obtained through acid hydrolysis of chitin, a linear polymer of a β-(1,4)-N-acetyl-D-glucosamine primarily extracted from the exoskeleton of crustaceans [17,21]. This polymer is a non-toxic, renewable, biodegradable, and biocompatible material that holds great potential for use in food applications [21].

Unlike the outer interface of DEs, the substitution of conventional emulsifiers with solid particles at the W_1_:O of DEs remains limited, and the formulation of Pickering DEs with both interfaces stabilized by solid particles remains even less studied [13,15]. Fat crystals and ethylcellulose are among the few solid particles employed in stabilizing the W_1_:O through a Pickering mechanism [10,13,22,23,24]. Recently, our research group synthesized myristic acid-functionalized silica nanoparticles (SNPs-C14), proving to be effective in stabilizing the W_1_:O of DEs containing chlorogenic acid and curcumin [25]. Silica has several characteristics that make it an attractive nanomaterial (mechanically stable, chemically inert, optically transparent, and fairly biocompatible). Amorphous silica is regarded as a safe additive in food applications, having been approved by major food safety authorities [26,27], with several studies supporting its application in food products [28]. In this context, considering the scarce research on the formulation of DEs stabilized exclusively by solid particles and the application of ChNCs for Pickering stabilization, the innovative aspect of this work is the formulation of Pickering DEs by employing ChNCs to stabilize the outer interface, as well as DEs with both interfaces stabilized by solid particles (ChNCs and SNPs-C14), as oral co-delivery matrices of curcumin and chlorogenic acid. These bioactive compounds were selected for encapsulation due to their interesting biological activities and their suitability as models for lipophilic and hydrophilic bioactive compounds, respectively [29,30]. Additionally, both compounds had been used in previous studies, allowing for a direct comparison of the results obtained in this work. This study aimed to evaluate the release behavior of curcumin and chlorogenic acid in DEs with a ChNC-stabilized outer interface, as well as in DEs where both interfaces were stabilized via the Pickering mechanism (ChNCs at the outer interface and SNPs-C14 at the inner interface), under in vitro gastrointestinal digestion conditions. Furthermore, both Pickering DEs were analyzed for their droplet size, ζ-potential, microscopic structure, encapsulation efficiency and stability, rheological behavior, and gravitational stability. Their characteristics were compared with those of DEs stabilized with conventional emulsifiers (PGPR at the inner interface and sodium caseinate/pectin at the outer interface).

## 2. Materials and Methods

### 2.1. Materials

Bioactive compounds were obtained from Xi’an Xin Sheng Bio-Chem Co. (Xi’an, China) for curcumin (total curcuminoids > 95%) and Sigma-Aldrich (Santiago, Chile) for chlorogenic acid (CA, purity 98%). Nutra Andes Ltd.a. (Valparaíso, Chile) was the supplier of linseed oil. Enzymes (pancreatin from porcine pancreas, P7545, 8 × USP specifications; pepsin from porcine gastric mucosa, P7012, 2500 AU/mg) and bile (porcine bile extract, B8631) for simulated in vitro digestion were acquired from Sigma-Aldrich (Santiago, Chile). Chitin derived from crustacean shells was sourced from AK Scientific (Union City, CA, USA). Grupo Blumos (Santiago, Chile) kindly provided high methoxy pectin (Aglupectin HS-RAM). Dimerco S.A. (Santiago, Chile) and Prinal S.A. (Santiago, Chile) supplied PGPR and sodium caseinate, respectively.

### 2.2. Chitin Nanocrystal (ChNC) Preparation

The ChNCs were obtained through acid hydrolysis [31] with slight modifications. The chitin (5 g) was weighed into a round-bottom flask. Then, 200 mL of HCl (3 M) was added, and the chitin suspension was boiled for 2 h using a heating mantle with a condenser attached to prevent gas loss. After boiling, the hydrolyzed chitin was allowed to sediment, and the settled material was collected. The concentrate was neutralized in distilled water using SnakeSkinTM dialysis tubing (3500 MQCO, 22 mm × 35 ft dry diameter) until the pH reached 4.5. Finally, the concentrate was freeze-dried (L200, Büchi, Flawil, Switzerland) and stored at room temperature until use.

### 2.3. Characterization of Chitin Nanocrystals (ChNCs)

#### 2.3.1. Size of ChNCs

An aqueous dispersion of ChNCs (1% *w*/*w*) was prepared and sonicated at 30 kHz frequency and 100% amplitude for 3 min using a UP100H ultrasonic processor (Hielscher, Teltow, Germany). A Zetasizer Nano-ZS (Malvern Instruments, Malvern, UK) was used to determine the hydrodynamic diameter of the nanocrystals by dynamic light scattering (DLS) at 25 °C.

#### 2.3.2. Wettability of ChNCs

The contact angle (ϑ_oil/water_) among the particles, water, and oil (which are the components of the emulsion interface) was measured with a contact angle goniometer (Ramé-hart 200-F4, Instrument Co., Succasunna, NJ, USA). Briefly, 500 mg of particles was compressed into dense discs using a manual press. Each disc was then fully immersed in linseed oil, and three drops of 5 µL of water were deposited on the disc. ImageJ software (version 1.54g) with the “Contact Angle” plugin was used to measure the contact angle.

#### 2.3.3. Microstructure of ChNCs

The microstructure of ChNCs was assessed using transmission electron microscopy (TEM; HT7700 microscope, Hitachi, Tokyo, Japan) under an acceleration voltage of 60 kV. The samples were prepared by applying a ChNC suspension droplet (5 µL) to a carbon-coated copper grid and allowing it to dry at room temperature.

The obtained TEM images were also analyzed to measure particle size (length and width) using the Levenhuk ToupView image software (version 4.1; Hangzhou, China). Three TEM images were analyzed, and 150 nanocrystals were measured per image. Equation (1) was used to calculate the volume mean diameter (D_4,3_) of ChNCs:

(1) $$D_{4,3}=\sum n_{n}l_{i}^{4}/\sum n_{n}l_{i}^{3}$$

where ni is the number of nanocrystals with length li.

#### 2.3.4. ζ-Potential of ChNCs

Laser Doppler velocimetry (Zetasizer Nano-ZS, Malvern Instruments, UK) was used to determine the ζ-potential of ChNC particles at 25 °C. A 0.1% (*w*/*w*) ChNC suspension using milli-Q was prepared and adjusted to various pH values (from 2 to 7). The suspensions were subjected to ultrasonication (30 kHz, 100% amplitude) for 5 min before measurement.

#### 2.3.5. X-Ray Diffraction

Small-angle X-ray scattering (SAXS) measurements were carried out using a SAXSPoint 2.0 system (Anton Paar, Graz, Austria), which features a microfocus copper X-ray source (Primus 100, Cu Kα = 1.54178 Å) operating at 50 kV and 100 μA, an ASTIX multilayer mirror for point collimation, and an Eiger R 1 M bidimensional detector (Dectris, Baden-Daettwil, Baden, Switzerland). A sample of ChNCs was placed between a Mylar^®^ film and a self-adhesive Kapton^®^ tape and analyzed without rotation. Data acquisition was performed at a detector distance of 116 mm, covering an angular range from 0 to 35°; the detector was aligned perpendicular to the beam, and the sample–detector distance was calibrated using a LaB_6_ standard. A single frame with an exposure time of 900 s was collected. The patterns were subsequently processed using the SAXSDrive software (version 2.01.224, Anton Paar, Austria). They were corrected for transmission effects, and the scattering contribution from the Mylar/Kapton films was subtracted to obtain the final background-corrected pattern. The patterns were reduced into one-dimensional scattering intensities as a function of the scattering vector magnitude q (q = (4π/λ)sin θ), where 2θ represents the total scattering angle).

### 2.4. Preparation of W_1_/O Emulsions

SNPs-C14 were synthesized and characterized according to a previous study [25] (D_4,3_ 156.6 ± 0.6 nm; ϑ_oil/water_ 123.7 ± 0.2°). PGPR or SNPs-C14 were used as stabilizers for simple W_1_/O emulsions (SE), resulting in SE-PGPR and SE-C14, respectively. An ultrasonic processor was used to formulate both emulsions (30 KHz, 100% amplitude) with sonication applied for 3 min in a 1 s on/1 s off cycle. The internal aqueous phase (W_1_, 20%) of both SEs contained 0.1% CA (*w*/*w*). The oil phase (80%) was composed of 0.3% curcumin (*w*/*w*), 4% SNPs-C14 (*w*/*w*) [25], and linseed oil (up to 100%). Linseed oil was selected due to its high content of α-linolenic acid, an essential fatty acid linked to numerous health benefits and a precursor to eicosapentaenoic and docosahexaenoic fatty acids [32].

### 2.5. Preparation of Pickering DE-ChNC and DE-ChNC-C14

In the case of both Pickering DEs, the external aqueous phase (W_2_) was composed of 6.7% ChNCs *w*/*w* (equivalent to 0.1 g of ChNCs per g of W_1_:O; [31]) dispersed in glycine buffer (0.25 M, pH 3.6) using an ultrasonic processor (30 kHz, 100% amplitude, 2 min), along with 0.1% chitosan (*w*/*w*) dispersed using a homogenizer (Omni GLH 850, OMNI International, Kennesaw, GA, USA) at 10,000 rpm for 5 min. DE-ChNC and DE-ChNC-C14 emulsions were obtained by adding the SE-PGPR or SE-C14 emulsion (40%) drop by drop, respectively, to the W_2_ (60%) with a homogenizer (GLH 850, OMNI International, USA). The osmolarity of both aqueous phases was measured with an osmometer (Osmometer M, Löser, Berlin, Germany) and adjusted with NaCl to minimize diffusion effects. The optimization of the homogenization time and speed for the formulation of DE-ChNC was conducted using a central composite design with star points. This study considered the homogenization time (between 1 and 15 min) and homogenization speed (from 5000 to 15,000 rpm) as independent variables (Table S1). In contrast, the dependent variables were the encapsulation efficiency (EE) of CA and the fraction of oil droplets exhibiting sizes smaller than 10 µm, expressed as a percentage (F_10_). To model the experimental data, a second-order regression equation was used (Equation (2)):

(2) $$Y=b_{0}+\sum_{i=1}^{2}b_{i}X_{i}+\sum_{i=1}^{2}b_{ii}X_{i}^{2}+\sum_{i=1}^{1}\sum_{j=i+1}^{2}b_{ij}X_{i}X_{j}$$

in which Y represents the estimated response; b_0_ is the intercept term; the b_i_, b_ii_, and b_ij_ values are the linear, quadratic, and interaction coefficients, respectively; X_i_ and X_j_, which are the levels of the independent variables with subscripts i and j, range from 1 to 2 (n = 2).

The Statgraphics Centurion 19 (version 19.6.05) software (Statgraphics Technologies Inc., The Plains, VA, USA) was used to perform the statistical analyses of the experimental design results. Using response surface methodology (RSM), the optimal conditions for each independent variable were established. Multiple optimization was carried out using the desirability function to maximize the EE of CA and minimize F_10_. DE-ChNC-C14 was also formulated using these optimized homogenization parameters, as ChNCs served as the stabilizer of the outer O:W_2_ interface.

A conventional double emulsion (control DE) was formulated for comparative purposes as previously described by our research group [25], using 6% PGPR (*w*/*w*) as the hydrophobic emulsifier for the inner interface and a blend of 0.5% sodium caseinate (*w*/*w*) and 3% pectin (*w*/*w*) as the hydrophilic emulsifier for the outer interface.

### 2.6. Characterization of DEs Formulated Under Optimal Homogenization Parameters

#### 2.6.1. Oil Droplet Size and Size Distribution, Microscopic Structure, Gravitational Stability, and ζ-Potential

The emulsions were refrigerated at 4 °C for 21 days, and the oil droplet size (represented by D_4,3_) and size distribution were determined periodically by light scattering with a particle analyzer (Mastersizer 2000^®^, Malvern Instruments Limited, Malvern, UK) according to Paredes-Toledo et al. [25]. Additionally, the F_10_ value was obtained from the droplet size distribution.

The microscopic structure of fresh DEs and those stored at 4 °C for 21 days was assessed by Confocal Laser Scanning Microscopy (CLSM; LSM 700, Zeiss, Jena, Germany), with image processing performed using the ZEN 2012 (version 3.6) software (Blue Edition, Carl Zeiss, Germany). Linseed oil was dyed with Nile Red fluorescent dye at a concentration of 0.01% (*w*/*w*). The experimental procedure was detailed in our previous study [25].

The gravitational stability of DEs during storage at 4 °C was evaluated using a Turbiscan (MA2000, Formulaction, Toulouse, France). The backscattering profiles were employed to determine the creaming index (CI) using Equation (3):

(3) $$CI=\frac{HS}{HE}\times 100$$

in which HS and HE indicate the serum layer height and the total emulsion height, respectively.

Laser Doppler velocimetry (Zetasizer Nano-ZS, Malvern Instruments, UK) was used to measure the ζ-potential of the emulsions over the course of the 21-day refrigerated storage. The DEs were diluted at a ratio of 1:2500 (*v*/*v*) in distilled water, with the pH adjusted from 2 to 7, and measured at 25 °C, as described by Paredes-Toledo et al. [25].

#### 2.6.2. Encapsulation Efficiency (EE) and Encapsulation Stability (ES)

The EE and ES of both bioactive compounds, curcumin and CA, were measured according to Paredes-Toledo et al. [25]. Briefly, the concentration of bioactives present in W_2_ of newly prepared samples and in W_2_ of DEs during storage at 4 °C was measured after gentle centrifugation (2400 g/15 min) using a UPLC (UltiMate 3000, Thermo Scientific, Waltham, MA, USA). The method reported by Qi et al. [33] was followed to measure the CA content, using a Symmetry C18 (4.6 × 250 mm column; 5 µm; Waters, Milford, MA, USA). The curcumin concentration was measured as described by Marczylo et al. [34], with separation performed on an Acquity BEH Shield RP18 (2.1 × 100 mm column, 1.7 µm; Waters, USA). Standard calibration curves were used to quantify both bioactive compounds (0.1–100 µg/mL; R^2^ = 0.99). The EE and ES of both bioactives were calculated using Equation (4):

(4) $$EE(\%)\;or\;ES\left(\%\right)=100-\frac{C_{w2}\times 100}{C_{t}}$$

where C_W2_ is the non-encapsulated CA or curcumin concentration recovered from W_2_ at day 0 or periodically during storage, and C_t_ is the initial total curcumin or CA concentration in the emulsions.

The loading capacity was determined as described by Li and Abbaspourrad [35] using Equation (5):

(5) $$LC\left(\%\right)=100\cdot \frac{m_{total}-m_{free}}{m_{DE}}$$

where m_total_ is the total mass of the bioactive compound added to the W_1_ phase (CA) or oil phase (curcumin), m_free_ is the mass of free CA or curcumin in the W_2_ phase after emulsion preparation, and m_DE_ is the total mass of the DE.

#### 2.6.3. Rheological Behavior

The rheological behavior of the emulsions was studied using a rheometer (Discovery Hybrid Rheometer, HR-1, TA Instruments, New Castle, DE, USA; 60 mm cone-plate geometry, 1°, 29 μm gap) at 25 °C. The apparent viscosity (Pa·s) was measured under increasing shear rates (0.01–1000 s^−1^). The linear viscoelastic region (LVR) was determined through dynamic amplitude sweeps, recording the storage modulus (G′) and loss modulus (G″) vs. shear stress (0.01 to 10 Pa) at 0.1 Hz. The viscoelastic properties of the samples were evaluated by frequency sweeps (0.1–10 Hz) conducted at a shear stress of 0.1 Pa (within the LVR), recording G′ and G″ vs. frequency.

### 2.7. Release of Curcumin, Chlorogenic Acid, and Fatty Acids During In Vitro Digestion

The emulsions formulated under optimal homogenization parameters underwent in vitro gastrointestinal digestion [36], simulating the conditions of the oral, gastric, and intestinal phases. Simulated salivary (SSF), gastric (SGF), and intestinal (SIF) fluids were prepared as reported by Brodkorb et al. [36], along with the enzyme concentrations used in the gastric and intestinal phases (2000 U pepsin/mL of gastric phase; 2000 U lipase activity in pancreatin/mL of intestinal phase). The amount of DE to be digested, the agitation conditions, and the incubation conditions (time and temperature) in each digestion phase were the same as described for DEs in a previous study [25]. After each phase of digestion, the structural integrity of DEs was assessed using an optical microscope (DM500, Leica Microsystems, Wetzlar, Germany), and the release of curcumin and CA was determined. For this, the aqueous phase was recovered by centrifuging the digesta (990 g for 5 min for the oral and gastric phases, and 7500 g for 60 min for the intestinal phase). The curcumin and CA concentrations were measured as detailed in Section 2.6.2. The bioaccessibility of curcumin and CA was determined using Equation (6).

(6) $$Bioaccessibilty\;\left(\%\right)=100-\left(C_{AD}/C_{i}\right)\cdot 100$$

where C_AD_ is the concentration of curcumin or CA in the micellar phase and C_i_ is the concentration of curcumin or CA in the undigested DEs

The free fatty acids (FFAs) generated by digestion were extracted [37] and methylated [25] to obtain the fatty acid methyl esters (FAMEs). These were analyzed by gas chromatography (7890B, Agilent Technologies, Santa Clara, CA, USA), following the experimental conditions described by Paredes-Toledo et al. [25]. To identify and quantify the major individual fatty acids, calibration curves constructed with FAMEs as external standards were used (C18:1, 0.01–10 mg/mL; C18:2, 0.01–10 mg/mL; C18:3, 0.01–20 mg/mL; R^2^ = 0.99 in all cases). The major FFA bioaccessibility was also determined using Equation (5).

### 2.8. Statistical Analysis

Experiments were performed in triplicate. Differences among samples were considered statistically significant at *p* ≤ 0.05 and were analyzed using ANOVA and Tukey’s multiple range test with Statgraphics Centurion 19 (version 19.6.05) software (Statgraphics Technologies Inc., USA).

## 3. Results and Discussion

### 3.1. Characterization of ChNCs

**Figure 1:**
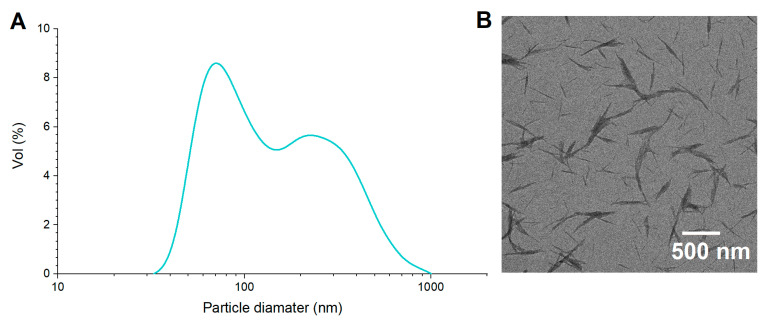
Particle size distribution of ChNCs obtained from DLS (**A**), and ChNC microstructure observed by TEM (**B**).

The crystalline nature of the ChNCs was confirmed by X-ray diffraction analysis (Figure S1). The diffraction pattern exhibited the characteristic reflections of α-chitin, with six intense crystalline diffraction peaks in the range of 5° < 2θ < 30°, corresponding to the 020, 021, 110, 120, 130, and 013 planes [31]. The ChNC particle size and size distribution, along with the contact angle ϑ_oil/water_ and microstructure, are shown in Figure 1. TEM images revealed a needle-like structure of ChNCs, with several rods joined in parallel, forming thicker particles (Figure 1B) as described by Zhu et al. [38]. The dimensions of the ChNCs, measured directly from TEM images, ranged from 50 to 900 nm in length and from 10 to 50 nm in width (Figure 1A), with D_4,3_ values of 505.9 ± 18.8 nm. When measured by DLS, ChNCs exhibited a bimodal size distribution, with sizes in the range of 30–100 nm and 100–1000 nm and a D_4,3_ value of 177.3 ± 2.6 nm (Figure 1A). The smaller size population detected by DLS corresponded to the ChNC width measured in the TEM images (Figure 1B), suggesting that the DLS method is not suitable for rod-shaped particles because it assumes spherical geometry. Other studies have formulated O/W Pickering emulsions using ChNCs with D_4,3_ values ranging from 250 to 350 nm (measured by DLS), achieving stable emulsions [39,40]. Ben Cheikh et al. [41] obtained ChNCs with similar sizes (length: 200–800 nm; width: 20–80 nm, measured from TEM images), which were used to stabilize O/W emulsions with a D_4,3_ value of 0.9 µm (soybean oil, 2% *w*/*w* ChNCs), exhibiting high gravitational stability over 30 days of storage.

The contact angle ϑ_oil/water_ was 73.8 ± 5.0°, similar to that of partially deacetylated ChNCs (~84.4°), which enabled the formulation of stable O/W Pickering emulsions [40]. The surface hydrophilicity (and thus the contact angle) of ChNCs is related to the proportion of hydrophobic N-acetyl groups, with higher proportions resulting in higher contact angle values [41]. The high contact angle observed in this study suggests a high proportion of N-acetyl groups in the ChNC structure, despite acid hydrolysis inducing partial deacetylation. With contact angle values close to 90°, ChNCs exhibited high oil/particle affinity, positioning them within the required range for O/W emulsions (15–90°; [42]) and indicating their potential to maintain the stability of the O:W_2_ interface of DEs.

### 3.2. Preparation and Characterization of DEs

#### 3.2.1. Optimization of Homogenization Parameters

Proper selection of the homogenization technique and processing parameters is essential for the formulation of Pickering emulsions, given that these factors directly affect the emulsion stability and its final properties [43]. To obtain Pickering emulsions with relatively small droplet sizes, it is necessary to overcome the desorption energy associated with solid particles through the mechanical forces generated during high shear rate emulsification. However, overly intense or prolonged emulsification methods can hinder the formation of PEs, resulting in high polydispersity in the final emulsion [13]. Therefore, in this study, the homogenization time and speed conditions to formulate the Pickering DEs were optimized using a statistical design.

Table S1 presents the experimental conditions defined by the statistical design for the preparation of DE-ChNC using a rotor-stator homogenizer, together with the values recorded for the response variables. The EE of CA ranged from 35.6% to 63.9%, whereas the F_10_ values were in the range of 8.8–50.5%. The corresponding ANOVA for the formulation of DE-ChNC emulsions is shown in Table S2. For the EE of CA, the linear effect of homogenization time was significant (*p* ≤ 0.05). In contrast, for F_10_, both the linear effect of homogenization speed and its interaction with homogenization time were significant (*p* ≤ 0.05). A total of 74.1% and 68.0% of the variability in the F_10_ and EE of CA data, respectively (R^2^ adjusted for degrees of freedom), was explained by the model. As the lack-of-fit test results were statistically non-significant in all cases (*p* > 0.05), the model was considered appropriate for describing the response variable data.

**Figure 2:**
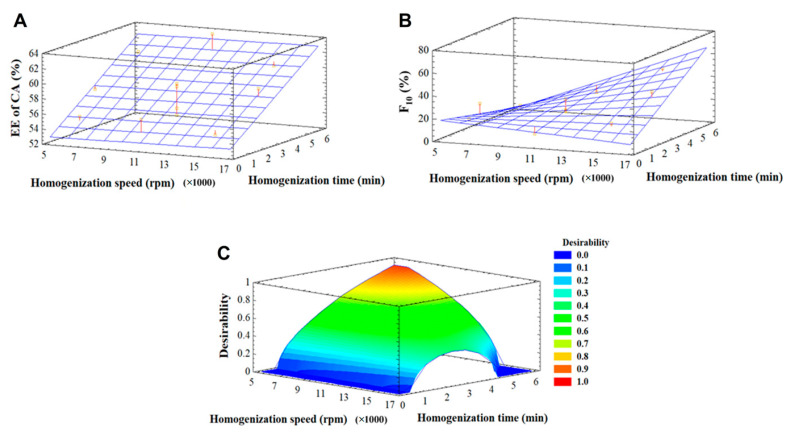
Response surface graphs depicting EE of CA (**A**), F_10_ (**B**), and desirability function (**C**) for the formulation of ChNC-stabilized DEs.

As shown in Figure 2A, homogenization time was a determining factor for the EE of CA, which increased with longer homogenization times (Figure 2A). However, the EE of CA was minimally affected by homogenization speed. For F_10_, increasing both the homogenization speed and time resulted in a marked increase in this parameter, as a higher energy input reduces the oil droplet size (Figure 2B). Therefore, the optimal homogenization conditions for DE-ChNC are likely to involve prolonged times (to increase the EE of CA) and relatively low speeds (to reduce F_10_). Figure 2C presents the desirability function applied to optimize both dependent variables for DE-ChNC by minimizing the F_10_ and maximizing the EE of CA. A maximum desirability value of 0.88 was obtained at a homogenization speed of 6028 rpm (rounded to 6000 rpm) and a homogenization time of 5.42 min. According to the model, these operating conditions would yield an EE of CA of 61.7% and an F_10_ value of 8.8%. These optimized parameters were then employed to prepare the DE from SE-C14, as its O:W_2_ was also stabilized with ChNCs.

#### 3.2.2. Oil Droplet Size and Distribution, Microscopic Structure, Gravitational Stability, and ζ-Potential

**Figure 3:**
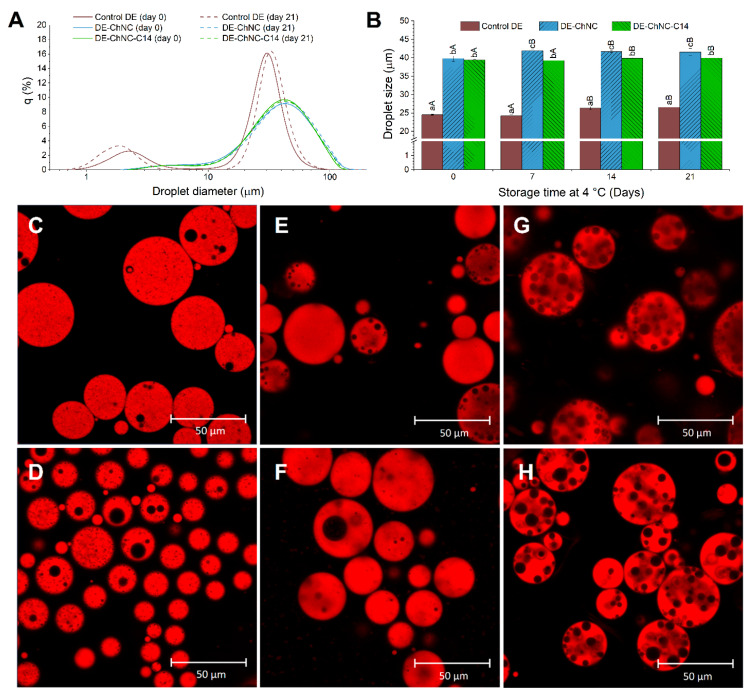
Oil droplet size distribution (**A**) and D_4,3_ values (**B**) of control DE, DE-ChNC and DE-ChNC-C14 over storage (4 °C/21 days). Significant differences (*p* ≤ 0.05) among emulsions at the same storage time are indicated by different lowercase letters (a–c), while significant differences (*p* ≤ 0.05) within the same sample over storage are indicated by different uppercase letters (A–B). CLSM images of control DE ((**C**): day 0 and (**D**): day 21), DE-ChNC ((**E**): day 0 and (**F**): day 21), and DE-ChNC-C14 ((**G**): day 0 and (**H**): day 21).

Figure 3 shows the size, size distribution, and microstructure of the DE-ChNC and DE-ChNC-C14 formulated under optimal parameters. Monitoring these characteristics over time provides insight into the emulsions’ stability during storage, enabling the assessment of potential destabilization mechanisms such as droplet coalescence and creaming.

The D_4,3_ values of the DE-ChNC and DE-ChNC-C14 were 39.8 ± 0.7 µm and 39.4 ± 0.2 µm, respectively. A minor rise (less than 2 µm) was observed in both emulsions after 7 and 14 days (*p* ≤ 0.05) and remained stable until day 21 of storage (Figure 3B). In previous studies [41,44], O/W Pickering emulsions with smaller oil droplets (ranging from 0.5 to 2.4 µm) were obtained using similar ChNCs at lower concentrations in the aqueous phase (0.35 to 5% vs. 6.7% *w*/*w* in this study). However, in those cases, Pickering emulsions were obtained by ultrasonication to achieve the smallest possible oil droplet size, whereas in this study, the F_10_ percentage was minimized in the experimental design, leading to larger oil droplets. The distribution of oil droplet sizes in DE-ChNC and DE-ChNC-C14 remained stable during the evaluated storage period (Figure 3A). The minimal changes observed in the evolution of the D_4,3_ values and size distribution indicate a lack of significant coalescence, suggesting good droplet stability over time. This stability during refrigerated storage suggests that the ChNCs likely formed a physical barrier at the outer O:W_2_ interface of these DEs, preventing oil droplet coalescence. Tzoumaki et al. [45] reported comparable findings, where no changes were observed in D_4,3_ during 30 days at room temperature, even with larger oil droplets (about 100 µm). This effect was ascribed to particles possessing the appropriate surface energy or contact angle with the interface, which enables them to effectively and irreversibly adsorb once attached to the interface. Much smaller oil droplets were obtained (24.5 ± 0.1 µm; *p* ≤ 0.05) when conventional emulsifiers were used to stabilize these DEs (control DE), which remained stable in size throughout the studied storage period, with only slight changes in the size distribution (Figure 3A,B). Since conventional emulsions do not involve solid particles, droplet size is not directly constrained by particle size but rather by factors such as the critical micelle concentration of the surfactant and the water/oil volume ratio. As a result, smaller oil droplets can be obtained with a comparable energy input during homogenization.

Consistent with the high stability observed in droplet size, a stable microstructure was maintained throughout the entire storage period in the Pickering DEs (Figure 3E,F and G,H for DE-ChNC and DE-ChNC-C14, respectively), showing no noticeable variations in the size of either the water or oil droplets, indicating the absence of significant coalescence. DE-ChNC-C14 exhibited a larger water droplet size than DE-ChNC, due to the use of SNPs-C14 to stabilize the water droplets, which are of much larger molecular size compared to the PGPR emulsifier molecules used to stabilize the water droplets in DE-ChNC [25]. Interestingly, when the outer interface was stabilized with conventional emulsifiers (control DE), the oil droplets became smaller, whereas the inner water droplets increased in size after 21 days of storage (Figure 3C,D), consistent with inner water droplet coalescence and their release to W_2_. This highlights the enhanced stability provided by the ChNCs in preventing the coalescence of both W_1_ and the oil droplets. Pickering DEs typically exhibit long-term microstructural stability compared to surfactant-stabilized systems due to the irreversible adsorption of solid particles at the interfaces, forming a steric barrier that prevents coalescence and Ostwald ripening [15,16]. In line with the results obtained in this study, the formation of Pickering DEs with long-term stability has been reported using other types of solid particles. For instance, emulsions stabilized with 2.5% and 3% OSA starch maintained their microstructure and a stable droplet size over 90 days of storage [46]. In another study, no increase in droplet size was observed during 20 days of storage in DEs stabilized with whey protein fibril–cellulose nanocrystal complexes at their external interface, indicating that coalescence did not occur [47].

**Figure 4:**
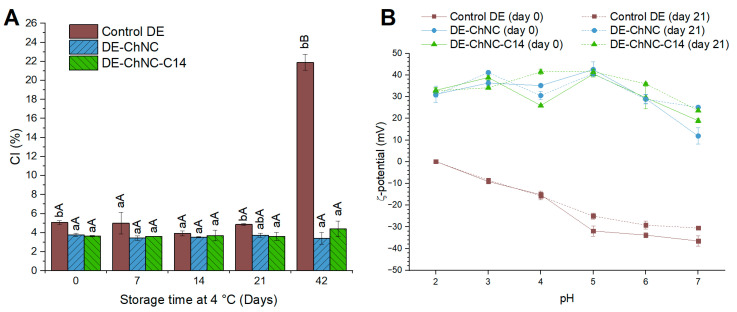
Creaming index of control DE, DE-ChNC, and DE-ChNC-C14 during storage (4 °C/21 days) (**A**). Significant differences (*p* ≤ 0.05) among emulsions at the same storage time are denoted by different lowercase letters (a–b), while significant differences (*p* ≤ 0.05) within the same sample over storage are represented by different uppercase letters (A–B). ζ-potential of control DE, DE-ChNC, and DE-ChNC-C14 at day 0 and day 21 across a pH range of 2–7 (**B**).

Consistent with the microscale stability, both the DE-ChNC and DE-ChNC-C14 exhibited high gravitational stability throughout storage (42 days at 4 °C), with CI values in the range of 3–7% (Figure 4A) and no significant variations (*p* > 0.05) throughout the storage period, despite the density difference between the W_1_/O droplets and the external aqueous phase, as well as their large droplet size. This stability may be attributed to the formation of a three-dimensional network by ChNCs within the W_2_ phase, which traps the oil droplets and increases the emulsion viscosity, thereby enhancing gravitational stability [41]. Additionally, the presence of chitosan dispersed in the W_2_ phase (0.1% *w*/*w*) further contributes to the increased viscosity, reinforcing the resistance to creaming. Furthermore, the anchoring of the ChNCs at the interface prevents coalescence, limiting droplet growth and thereby reducing creaming. Typically, Pickering emulsions are prone to creaming due to their large droplet size, unless thickening agents are employed in the external aqueous phase [16] or the particles stabilizing the outer interface can form networks, as in the case of ChNCs. The high gravitational stability of the ChNC-stabilized O/W emulsions has been described in previous works [45], in which O/W emulsions (D_4,3_ ~10 µm) containing only 1% ChNCs (*w*/*w*) did not exhibit creaming during 6 months at room temperature. Other solid particles, such as whey protein fibril–cellulose nanocrystal complexes or gliadin nanoparticles [13,47], have enabled the formation of DEs with high resistance to creaming when used to stabilize the outer interface of DEs due to the formation of three-dimensional networks in the continuous phase that prevent droplet movement. Notably, stabilization of the outer interface with ChNCs for both DE-ChNC and DE-ChNC-C14 provided higher gravitational stability after 42 days of storage (CI of 3.4 ± 0.6% and 4.4 ± 0.8%, respectively) compared to the control DE, where the inner interface was stabilized with PGPR and the outer interface with sodium caseinate and pectin (CI of 21.9 ± 0.8%). This suggests that the stabilization mechanism used at the outer interface of DEs serves as a decisive factor in their gravitational stability, while stabilizing the inner interface with SNP-C14 particles does not contribute to enhanced stability.

The ζ-potentials of the DE-ChNC, DE-ChNC-C14, and control DE are shown in Figure 4B. Both Pickering DEs showed a positive charge, ranging from 26 to 43 mV within the pH range of 2–6, consistent with the positive ζ-potential values of ChNCs (Figure S2). This behavior is attributed to the protonation of primary amine groups due to the partial deacetylation of chitin in an acidic medium [39]. DE-ChNC and DE-ChNC-C14 exhibited pH values of 3.73 ± 0.03 and 4.09 ± 0.01, respectively, suggesting intense electrostatic repulsion among the emulsion droplets resulting from the high ζ-potential values at these pH levels (Figure 4B), which contributes to the droplet size and gravitational stability described above. When the pH levels exceeded 6, the ζ-potential of both the ChNCs and Pickering DEs diminished because of the deprotonation of the amine groups in ChNCs, reaching values of approximately 12 mV and 19 mV in DE-ChNC and DE-ChNC-C14, respectively, at pH 7. This suggests the reduced stability of these colloidal systems at the intestinal digestion pH as a result of decreased electrostatic repulsion.

In contrast to DE-ChNC and DE-ChNC-C14, the control DE showed notable differences across the studied pH range, with ζ-potential values decreasing from 0.1 to −36.3 mV over the pH range of 2 to 7. A similar relationship between the ζ-potential and pH has also been observed in O/W emulsions where a blend of sodium caseinate and high methoxy pectin served as the emulsifier [48]. This behavior suggests significant differences in the stability of DEs when subjected to gastrointestinal conditions, as at pH 2 (corresponding to the gastric phase), no electrostatic repulsion was observed between the oil droplets in the control DE (Figure 4B). All DEs exhibited similar ζ-potential values after 21 days of storage across the studied pH range, indicating good stability arising from the electrostatic repulsion between oil droplets.

#### 3.2.3. Loading Capacity (LC), Encapsulation Efficiency (EE), and Stability (ES)

The LC of CA in DE-ChNC and DE-ChNC-C14 was 0.0036 ± 0.0002% and 0.0073 ± 0.0002%, respectively. Meanwhile, the LC of curcumin in DE-ChNC and DE-ChNC-C14 was 0.0875% ± 0.0001% and 0.0874 ± 0.0003%, respectively, with no significant differences (*p* > 0.05). The LC of hydrophilic and lipophilic bioactive compounds in DEs is strongly influenced by the mass fraction of the W_1_ and oil phases in the emulsion (8% *w*/*w* and 32% *w*/*w*, respectively, in both DE-ChNC and DE-ChNC-C14), as well as the solubility of the bioactive compounds in their respective phases [35].

The EE of CA was 45.0 ± 2.7% and 91.4 ± 0.9% for DE-ChNC and DE-ChNC-C14, respectively (Table 1). Regarding the ES of CA, it remained constant in DE-ChNC until day 14 of storage (38–45%; *p* > 0.05) and significantly decreased at day 21 (32.9%; *p* ≤ 0.05). For DE-ChNC-C14, the ES of CA decreased to 52.0 ± 3.8% (*p* ≤ 0.05) on day 7 of storage and remained constant (*p* > 0.05) until the end of the storage (day 21). The control DE showed a significantly lower EE of CA (26.0 ± 0.1%) compared to the Pickering DEs and exhibited slight variations throughout the storage period (ES 24.9 ± 0.5% at day 21). The high EE and ES of Pickering DEs for hydrophilic compounds, such as anthocyanin and sucrose, compared to conventional DEs, has been ascribed to the irreversible anchoring of stabilizing particles at the interfaces, preventing droplet coalescence and diffusion between phases [10].

Comparing both Pickering DEs, DE-ChNC-C14 exhibited a significantly higher (*p* ≤ 0.05) EE and ES of CA than DE-ChNC. These differences suggest that even though the mechanism of stabilization at the inner interface did not influence the macro- or microstructural stability of ChNC-stabilized DEs (Figure 3 and Figure 4A), it played a key role in preventing the release of CA from W_1_ to W_2_. In contrast with PGPR, the SNPs-C14 are irreversibly anchored at W_1_:O, establishing a steric barrier that hinders the CA release from W_1_ during homogenization and storage. The use of solid particles (ChNCs and SNPs-C14) at both interfaces of DEs further increased the EE of CA (91.4 ± 0.9%) compared to the use of only SNP-C14 particles at the inner interface (81.3 ± 2.1%) [25], indicating that the barrier formed by the ChNCs at O:W_2_ also plays a role in the retention of CA. In other studies, high EE values of anthocyanin (85–97%) have been achieved in DEs where only the O:W_2_ was stabilized via the Pickering mechanism, using kafirin nanoparticles or modified quinoa starch granules, while the W_1_:O was stabilized with PGPR as in the case of DE-ChNC [49,50]. However, in our study, achieving similar values also required stabilizing the W_1_:O with SNP-C14 particles. This may be due to the lower efficiency of ChNCs in retaining CA compared to these other colloidal particles and/or the different nature of the encapsulated hydrophilic bioactive compound and its ability to migrate through the oil phase.

The EE and ES of curcumin are also shown in Table 1. Although the curcumin EE was over 90% in all the samples, the control DE showed higher (*p* ≤ 0.05) values than the Pickering DEs. After 21 days of storage, the ES of curcumin remained higher in the control DE (98.2%; *p* ≤ 0.05) than in the DE-ChNC and DE-ChNC-C14 (88.6% and 85.1%, respectively). These differences, along with the slight release of curcumin in Pickering DEs during storage, may be ascribed to the formation of complexes between the bioactive compound and the ChNCs through hydrogen bonding [51], promoting the release of curcumin from linseed oil to the W_2_ phase. Despite this, these Pickering DEs exhibited a high capacity for encapsulating curcumin (EE: 91%, ES at day 21: 85–88%), comparable to the values reported in other studies, both for conventional DEs (EE: 88%; [4]) and for Pickering DEs, such as those stabilized with sugar beet pectin–bovine serum albumin nanoparticles at the O:W_2_ interface and PGPR in W_1_:O (EE: 84%, ES: at day 14 75%; [52]).

#### 3.2.4. Rheological Behavior

**Figure 5:**
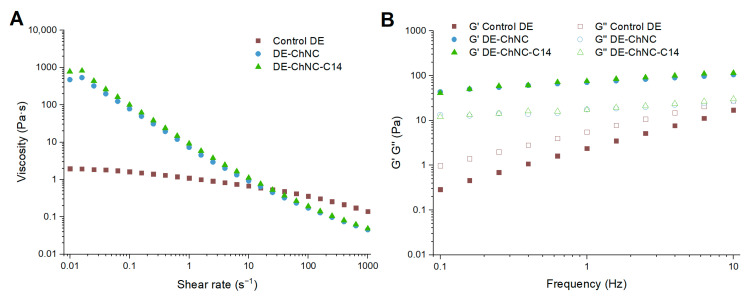
Flow behavior (**A**) and frequency sweeps (**B**) of control DE, DE-ChNC, and DE-ChNC-C14.

Figure 5A exhibits the flow behavior of DE-ChNC and DE-ChNC-C14. The apparent viscosity significantly decreased in both DEs with higher shear rates, exhibiting strong shear-thinning behavior regardless of the stabilization method at the inner interface. Nevertheless, both DEs exhibited a slight viscosity increase at low values of shear rate. This might be due to the quick rearrangement of the ChNC network occurring right after intense agitation and placing the sample in the rheometer [45]. In contrast, the control DE exhibited moderate shear-thinning flow behavior, with much lower apparent viscosity values (~2 Pa·s) at low shear rates (Figure 5A). Both Pickering DEs showed high values of apparent viscosity at low shear rates, (500–900 Pa·s), attributed to the formation of a three-dimensional network by the ChNCs not adsorbed at the interface [45] and the contribution of chitosan dispersed in the W_2_ phase. This viscosity decreased to 0.05 Pa·s as the shear rate increased from 0.02 s^−1^ to 1000 s^−1^, consistent with the shear-thinning behavior of Pickering emulsions. This behavior may be ascribed to the fact that, with increasing shear rate, oil droplets are forced to align with the flow direction, minimizing their interactions with ChNCs and gradually disrupting the ChNC–oil droplet network, ultimately leading to a reduction in viscosity [41]. The increased viscosity of DEs stabilized with ChNCs at low shear rates could slow the release of the bioactive compounds, particularly chlorogenic acid, contributing to the higher encapsulation stability (ES) observed in stored samples.

Regarding the viscoelastic properties of the samples, the G′ modulus exceeded G″ throughout the studied frequency domain in both Pickering DEs, showing a gel-like behavior (Figure 5B). Similar behavior has been observed in ChNC-stabilized O/W emulsions, where the network formed by ChNCs in the W_2_ phase and between emulsion droplets results in gel-like behavior, enhancing the emulsion stability [45,53]. Furthermore, a slight increase was observed in both moduli with increasing frequency values, suggesting a weak frequency dependence in the system characteristic of emulsions with moderately developed gel networks [45], similar to that reported for O/W emulsions in which stabilization was achieved using 0.1% (*w*/*w*) ChNCs [45]. In contrast, the control DE (Figure 5B) exhibited lower G′ and G″ values and a viscous behavior across the entire frequency range studied, along with a greater dependence of both moduli on frequency. This behavior is attributed to the absence of a network structure in the control DE, where pectin molecules are dispersed. Upon shearing, pectin molecules relax quickly due to their high molecular mobility, resulting in minimal stress accumulation, particularly at low frequencies [54].

### 3.3. Release of Curcumin, Chlorogenic Acid, and Fatty Acids During In Vitro Digestion

**Figure 6:**
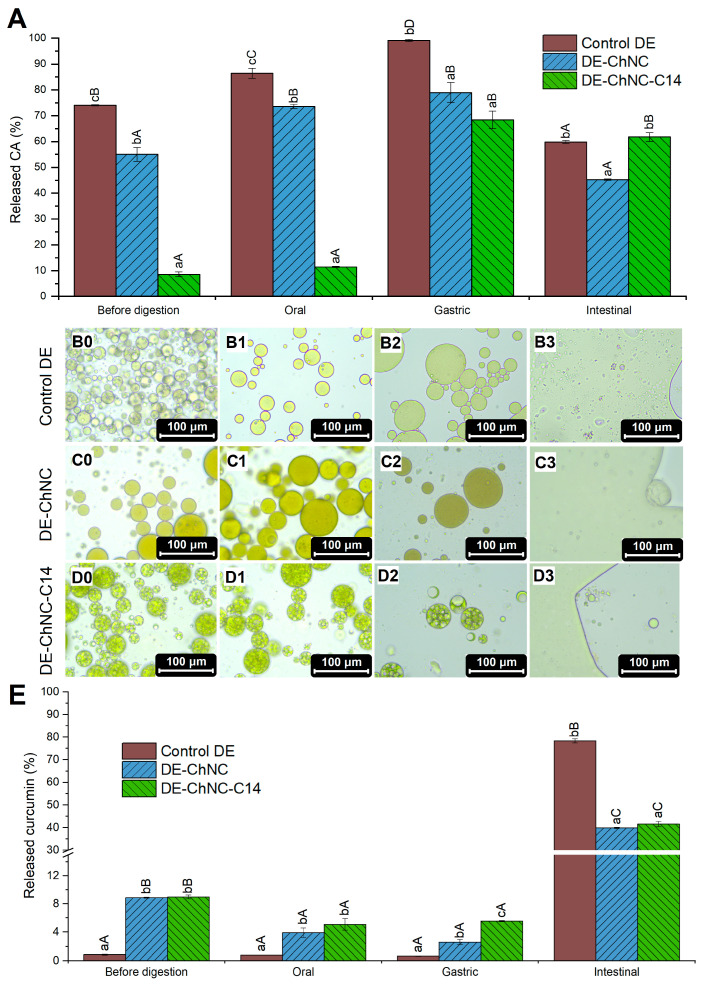
Release of CA (**A**) and curcumin (**E**) from control DE, DE-ChNC, and DE-ChNC-C14 before and after each phase of in vitro gastrointestinal digestion. Microstructure of DEs ((**B**): Control DE, (**C**): DE-ChNC, and (**D**): DE-ChNC-C14) before (0), and after oral (1), gastric (2), and intestinal (3) phase. Significant differences (*p* ≤ 0.05) among DEs at the same phase of digestion are indicated by different lowercase letters (a–c), whereas significant differences (*p* ≤ 0.05) among digestion phases for the same sample are indicated by different uppercase letters (A–D).

Figure 6 shows the release of CA and curcumin after the three phases of gastrointestinal digestion, along with the structural integrity of DEs. The release of CA from DE-ChNC and DE-ChNC-C14 during the oral phase was 18.6% and 2.9%, respectively, with total releases of 73.6 ± 0.8% and 11.4 ± 0.3% (Figure 6), taking into account the percentage of CA that was free in W_2_ before digestion according to the EE values (Table 1). Since both Pickering DEs showed similar microstructures before and after the oral phase (Figure 6C1,D1), the osmotic pressure difference between the W_1_ phase (310 mOsm/Kg) and the SSF (39 mOsm/Kg) is likely the primary cause of the CA release. After the completion of the oral phase, the CA released was lower (*p* ≤ 0.05) in DE-ChNC-C14 in comparison with DE-ChNC, indicating that the presence of SNPs-C14 in these DEs contributed significantly to preventing the release of the bioactive compound despite the previously mentioned osmotic pressure difference. During the gastric phase, approximately 5.4% and 57.0% additional CA was released from DE-ChNC and DE-ChNC-C14, respectively (Figure 6). These Pickering DEs have several features that ensure their stability in the stomach, such as the resistance of ChNCs to hydrolysis by pepsin, the strong electrostatic repulsion between oil droplets at stomach pH (pH 2) (Figure 4B), and, in the case of DE-ChNC-C14, the stability provided by SNPs-C14 at the inner interface. Accordingly, no evident changes were observed in the microstructure during this phase (Figure 6C2,D2), and both Pickering DEs retained their typical multicompartmentalized structure without any noticeable water droplet loss. Other studies have also described the high stability of the ChNC-stabilized O/W emulsion’s microstructure up to the gastric phase of simulated digestion [55]. However, despite these stabilizing factors, a significant CA release from W_1_ was found in DE-ChNC-C14 during gastric digestion, likely as a result of the osmotic gradient between W_1_ and the SGF. Nevertheless, the total percentage of CA released after gastric digestion was lower than that observed in DE-ChNC (*p* ≤ 0.05).

In the case of CA encapsulated in the control DE, approximately 15% was released during the oral phase, and the remaining encapsulated CA underwent complete release during gastric digestion (Figure 6A). In addition to the osmotic gradient between W_1_ and the digestive fluids, other factors may facilitate the CA release from W_1_ in this DE, such as the breakdown of pectin–caseinate complexes established at the outer interface in the oral phase pH (pH 7) and the susceptibility of caseinate to hydrolysis by pepsin in the gastric phase [25], resulting in the coalescence observed in this phase (Figure 6B2).

The collapse of the Pickering DEs observed after the intestinal digestion suggests the complete release of the CA that remained encapsulated upon completion of the gastric stage (Figure 6C3, D3), whereas in the control DE, CA was entirely released during gastric digestion. However, the bioaccessible fraction of CA after the intestinal phase was 45.2 ± 0.3% in DE-ChNC and approximately 60% for DE-ChNC-C14 and the control DE (61.8 ± 1.7% and 59.9 ± 0.6%, respectively) (Figure 6A). This may be due to the exposure of CA to intestinal conditions (pH 7), which could lead to the partial degradation of the bioactive compound, as it is unstable at this pH [56], resulting in lower bioaccessibility values. Furthermore, the lower bioaccessibility for CA observed in DE-ChNC may be attributed to electrostatic interactions between chitin, positively charged at pH 7 (Figure 4B), and the carboxyl group of chlorogenic acid, negatively charged at this pH (pKa 3.35) [57], reducing the amount of CA in the micellar phase. However, this behavior was not detected in DE-ChNC-C14, suggesting that SNPs-C14 may significantly contribute to the gradual CA release throughout all digestion phases, thereby enhancing the bioaccessibility despite these potential interactions. Additionally, the high values found for the control DE (59.9 ± 0.6%) could be associated with the presence of sodium caseinate, used as an emulsifier. Previous findings within this framework suggest that protein concentrations between 0.12% and 0.73% *w*/*w* resulted in CA bioaccessibility values of around 40% and 52%, respectively [58].

**Figure 7:**
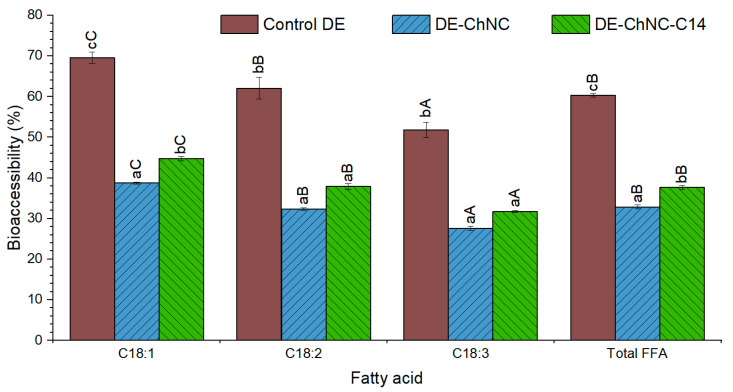
Bioaccessibility of total and major individual FFAs (%) from linseed oil in control DE, DE-ChNC, and DE-ChNC-C14. Significant differences (*p* ≤ 0.05) among samples for the same fatty acid are indicated by different lowercase letters (a–c), while differences (*p* ≤ 0.05) among fatty acids within the same emulsion are represented by different uppercase letters (A–C).

Throughout the oral and gastric phases, curcumin release remained under 10% in all DEs (Figure 6E), a value that is significantly lower than that of CA in these digestion stages, likely due to curcumin’s hydrophobicity. The amount of curcumin released from the Pickering DEs was higher than that of the control DE, which may be due to the ChNC/curcumin interactions described above, as non-adsorbed ChNC/curcumin complexes may migrate toward the SSF and SGF. During the intestinal phase, pancreatin lipases catalyzed the hydrolysis of linseed oil triglycerides, the main component of the oil phase of emulsions. Consequently, curcumin solubilized in the oil was released and subsequently incorporated into mixed micelles made up of fatty acids and bile salts. Both Pickering DEs, DE-ChNC and DE-ChNC-C14, exhibited relatively high curcumin bioaccessibility values (39.9 ± 0.3% and 41.5 ± 1.2%, respectively; Figure 6E), significantly higher than that of curcumin dispersed in water (6.9 ± 0.4%). However, these values were lower than those of the control DE (78.4 ± 0.9%) and previous studies where curcumin was co-encapsulated with hydrophilic bioactives in DEs stabilized with conventional emulsifiers (70–80%) [4,25] or other colloidal particles at the outer interface, including sugar beet pectin–bovine serum albumin nanoparticles (~53%) [52] and whey protein fibril–cellulose nanocrystal complexes (~68%) [47]. This lower bioaccessibility in DE-ChNC and DE-ChNC-C14 may result from the interaction of ChNCs and curcumin, leading to complex formation by hydrophobic interactions and hydrogen bonding [51], reducing the amount of curcumin available in the micellar phase. Additionally, the reduced curcumin release in DE-ChNC and DE-ChNC-C14 at the intestinal level may also be related to the fact that the ChNCs appear to interfere with lipid digestion, which leads to a lower release of fatty acids and fewer mixed micelles available for curcumin solubilization. ChNCs may not only hinder the access of lipases and bile salts by forming a steric barrier around oil droplets but also promote oil droplet flocculation, reducing the surface area available for lipolysis. Moreover, ChNCs can reduce lipase activity and interact with anionic bile salts, preventing their adsorption onto oil droplet surfaces [18,19,20,59]. According to this latest hypothesis, the bioaccessibility of total free fatty acids in this study was 33–38% for Pickering DEs (Figure 7), which is significantly lower (*p* ≤ 0.05) when compared to the control DE, in which the outer interface was stabilized with sodium caseinate (60.3 ± 0.4%). The same trend was observed in the bioaccessibility of the main individual fatty acids (Figure 7), with higher values (*p* ≤ 0.05) in the control DE than in the Pickering DEs. Moreover, a decline in bioaccessibility values was evident in all DEs as the unsaturation level of fatty acids rose. This trend has been reported in previous studies on DEs formulated with linseed oil and has been ascribed to the higher susceptibility of fatty acids with a lower degree of unsaturation to lipase hydrolysis, facilitated by the increased distance between the ester bond and the first unsaturation in the fatty acid chain, together with their more efficient incorporation in mixed micelles, which enhances their bioaccessibility [60].

Several studies have demonstrated that lipid digestion is lower in ChNC-stabilized O/W emulsions compared to conventional emulsions stabilized with whey protein isolate, sodium caseinate, or Tween 80 [18,20]. Moreover, lipid digestion is further inhibited in a concentration-dependent manner as the ChNC concentration increases [19]. Consequently, the bioaccessibility of lipophilic bioactives including β-carotene and vitamins, is significantly reduced [18,19,20].

## 4. Conclusions

Double emulsions were successfully stabilized using chitin nanocrystals (ChNCs) at the outer interface and myristic acid-functionalized silica nanoparticles (SNPs-C14) at the inner interface. The use of ChNCs at the outer interface of DEs enhanced the micro- and macrostructural stability, preventing oil droplet coalescence and creaming during storage, as the ChNCs irreversibly anchored at this interface and increased the emulsion viscosity. However, stabilizing the inner interface with SNPs-C14 was also necessary to achieve high CA retention, both during homogenization to form the double emulsion and throughout storage. Additionally, SNPs-C14 played an important role during gastrointestinal digestion, allowing a gradual release of CA throughout the three phases, as they provided a physical barrier that prevented the release of inner water droplets and delayed the release of CA due to osmotic gradients. Intestine-specific release of curcumin was achieved with both Pickering DEs. Relatively high curcumin bioaccessibility values were obtained despite the ChNCs slowing down triglyceride hydrolysis and/or curcumin solubilization during the intestinal phase. According to these results, ChNCs/SNPs-C14-stabilized DEs may hold great potential for the concurrent oral delivery of oil-soluble curcumin and water-soluble CA.

## Figures and Tables

**Table 1:** Chlorogenic acid (CA) and curcumin encapsulation efficiency (EE) and stability (ES) during 21 days of storage at 4 °C.

		Encapsulation Efficiency (EE) and Stability (ES) (%)			
	Double Emulsion (DE)	Day 0	Day 7	Day 14	Day 21
CA	Control DE	26.0 ± 0.1 ^aA^	31.7 ± 0.8 ^aB^	32.6 ± 0.3 ^aB^	24.9 ± 0.5 ^aA^
	DE-ChNC	45.0 ± 2.7 ^bB^	38.6 ± 3.6 ^aAB^	38.1 ± 1.1 ^aAB^	32.9 ± 0.6 ^bA^
	DE-ChNC-C14	91.4 ± 0.9 ^cB^	52.0 ± 3.8 ^bA^	54.0 ± 3.5 ^bA^	54.9 ± 3.0 ^cA^
	Double emulsion (DE)	Day 0	Day 21		
Curcumin	Control DE	98.5 ± 0.2 ^bA^	98.2 ± 0.1 ^cA^		
	DE-ChNC	91.1 ± 0.1 ^aB^	88.6 ± 0.2 ^bA^		
	DE-ChNC-C14	91.0 ± 0.3 ^aB^	85.1 ± 1.0 ^aA^		

Control DE: Double emulsion stabilized with polyglycerol polyricinoleate at the inner interface and sodium caseinate-pectin at the outer interface. DE-ChNC: Double emulsion stabilized with polyglycerol polyricinoleate at the inner interface and chitin nanocrystals at the outer interface. DE-ChNC-C14: Double emulsion stabilized with myristic acid-functionalized silica nanoparticles at the inner interface and chitin nanocrystals at the outer interface. Significant differences (*p* ≤ 0.05) among samples at the same storage time are indicated by different lowercase letters (a–c), while significant differences (*p* ≤ 0.05) at different storage times within the same sample are represented by different uppercase letters (A–B).

## Data Availability

Data will be available on request.

## Supplementary Materials

The following supporting information can be downloaded at: https://www.mdpi.com/article/10.3390/pharmaceutics17040521/s1, Figure S1: X-ray diffraction (XRD) analysis of the prepared ChNCs; Figure S2: ζ-potential of ChNCs and sodium caseinate (NaCas) + pectin (hydrophilic emulsifiers of control DE) at pH range 2–7; Table S1: Central composite experimental design with a star point for the preparation of DE-ChNC; Table S2: Analysis of variance (ANOVA) for the formulation of DE-ChNC.

## Abbreviations

The following abbreviations are used in this manuscript:

DEs	Double emulsions
SE	Simple W_1_/O emulsion
F_10_	Fraction of oil droplets exhibiting sizes smaller than 10 µm
FFAs	Free fatty acids
SSF	Simulated salivary fluid
SGF	Simulated gastric fluid
SIF	Simulated intestinal fluid
D_4,3_	Volume mean diameter
ϑ_oil/water_	Contact angle among the particles, water, and oil
CA	Chlorogenic acid
W_1_	Internal aqueous phase
W_2_	External aqueous phase
W_1_:O	Inner interface
O:W_2_	Outer interface
ChNCs	Chitin nanocrystals
SNPs-C14	Myristic acid-functionalized silica nanoparticles
DE-ChNC	Double emulsion stabilized with polyglycerol polyricinoleate at the inner interface and chitin nanocrystals at the outer interface.
DE-ChNC-C14	Double emulsion stabilized with myristic acid-functionalized silica nanoparticles at the inner interface and chitin nanocrystals at the outer interface
LC	Loading capacity
EE	Encapsulation efficiency
ES	Encapsulation stability
DLS	Dynamic light scattering
CLSM	Confocal laser scanning microscopy
TEM	Transmission electron microscopy
