# Supplementary material for: Pickering Double Emulsions Stabilized with Chitin Nanocrystals and Myristic Acid-Functionalized Silica Nanoparticles for Curcumin and Chlorogenic Acid Co-Delivery
Source: Pharmaceutics. 2025 Apr 16;17(4):521. doi: 10.3390/pharmaceutics17040521 (PMC12030632; doi:10.3390/pharmaceutics17040521)
Supplement: Supplementary file 1 [file pharmaceutics-17-00521-s001.zip › pharmaceutics-3536635-supplementary.pdf]

Table S1: Central composite experimental design with a star point for the preparation of DE-ChNC

DE	Homogeneization speed (rpm)	Homogeneization time (min)	EE of CA (%)	F_{10} (%)
1	11000	0.58	55.7 ± 0.6	8.8 ± 0.2
2	16000	3.00	59.1 ± 1.2	39.6 ± 0.5
3	6000	1.00	55.1 ± 0.9	15.9 ± 1.2
4	11000	5.42	63.9 ± 1.5	23.4 ± 0.8
5	6000	5.00	61.1 ± 2.1	9.5 ± 0.3
6	15000	1.00	54.1 ± 1.3	20.1 ± 0.5
7	15000	5.00	60.7 ± 0.8	50.5 ± 1.1
8	5000	3.00	57.3 ± 0.4	19.7 ± 0.6
9	11000	3.00	55.6 ± 0.8	28.2 ± 0.9
10	11000	3.00	58.5 ± 1.4	18.0 ± 0.4
11	11000	3.00	58.9 ± 1.2	17.8 ± 0.7
12	11000	3.00	54.8 ± 0.9	18.8 ± 0.6

DE: Double emulsion. EE of CA: Encapsulation efficiency of chlorogenic acid. F_{10}: Fraction of oil droplets smaller than 10 μm.

Table S2: Analysis of variance (ANOVA) for the formulation of DE-ChNC

	DE-ChNC	
	**Coefficient**	***p* value**
**EE of CA (%)**		
Constant	52.7618	
Homogenization speed	$2.49 \cdot 10^{-5}$	0.8947
Homogenization time	1.6254	0.0252*
Lack of fit		0.7694
R^2 adjusted by d.f.		68.0
**F_{10} (%)**		
Constant	22.3717	
Homogenization speed	$-8.44 \cdot 10^{-4}$	0.0135*
Homogenization time	−7.7264	0.0511
Homogenization speed x homogenization time	$1.02 \cdot 10^{-3}$	0.0351*
Lack of fit		0.3397
R^2 adjusted by d.f.		74.1

* significative ($p \leq 0.05$). DE-ChNC: Double emulsion stabilized with polyglycerol polyricinoleate (PGPR) at the inner interface and chitin nanocrystals (ChNC) at the outer interface. EE of CA: Encapsulation efficiency of chlorogenic acid. F_{10}: fraction of oil droplets smaller than 10 μm. d.f.: degrees of freedom.

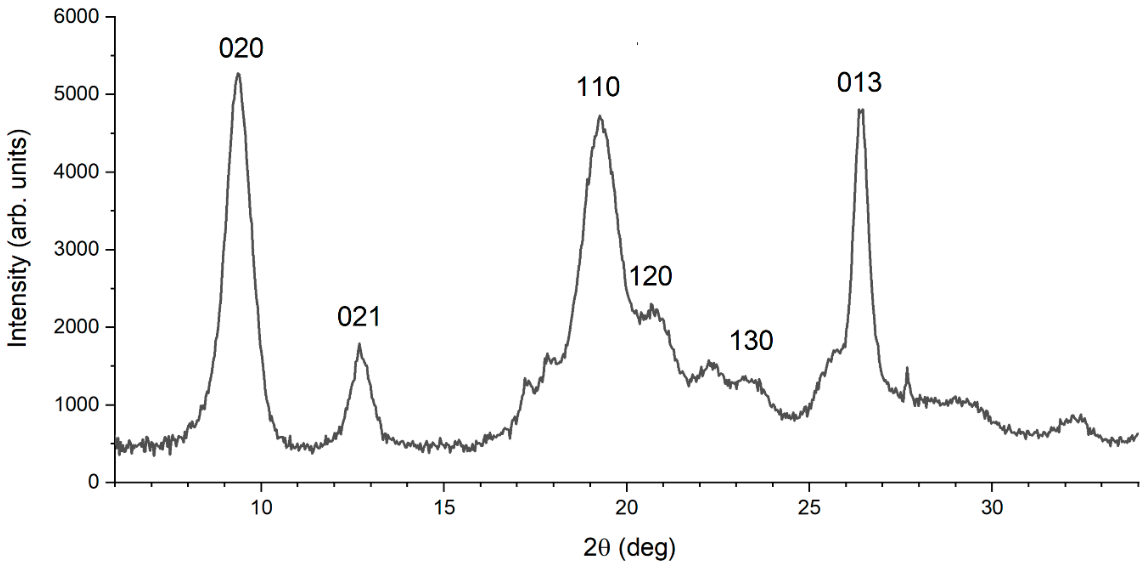

Figure S1: X-ray diffraction (XRD) analysis of the prepared ChNC.

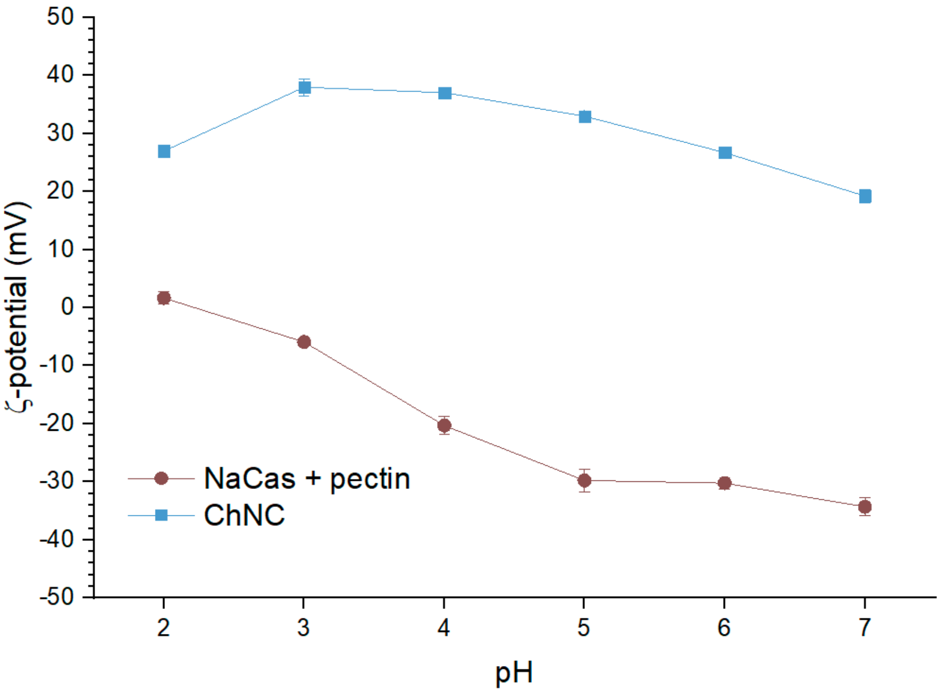

Figure S2: ζ-potential of ChNC and sodium caseinate (NaCas) + pectin (hydrophilic emulsifiers of control DE) at pH range 2–7.
